# A Clinical Lecture on Pneumonia

**Published:** 1893-11

**Authors:** A. Jacobi

**Affiliations:** Professor of Diseases of Children, College of Physicians and Surgeons, New York


### A CLINICAL LECTURE ON PNEUMONIA.

BY A. JACOBI, M. D.,

Professor of Diseases of Children, College of Physicians and Surgeons, New York.

---

The history states that this child, about eighteen months old, has had whooping-cough since the week before Christmas. During the past three weeks there has been some dyspnœa, elevation of the temperature, cough with expectoration of mucus.

We find a little dulness over the right upper lobe anteriorly and in the right axilla. I may repeat the fact, because of its importance, that in a large number of cases of lobular pneumonia in children you find dulness in the axillary line when it is not to be found anywhere else. Remember that you must percuss gently in order to avoid vibration from organs at a distance. There are rales over the entire chest. I believe I remind you of only that which you know when I say that pneumonia in children is different usually from the form seen in adults. The common form in adults is so-called croupous or fibrinous pneumonia, while in children it is usually the lobular or broncho-pneumonia. That means the pneumonia is the result of what originally was a simple bronchial catarrh. In most of the cases, say two-thirds of them, the process is this: Small bronchi are obstructed with viscid mucus; behind this obstacle the air-cells are collapsed, and the space which formerly was occupied by the air-cells must be filled by something else, namely, dilated blood-vessels. Thus you have congestion, and from that you have exudation. The effusion is localized, while the catarrh may exist over a hundred bronchi. The mucous obstacles may also exist in numerous places, giving rise to many pneumonic centers or lobular pneumonia. This condition may exist and yet give very little dulness on percussion, and still less bronchial or tublar respiration. In a number of cases, however, when you do not find bronchial respiration, you still find bronchophony. Therefore the crying of a baby is not at all an obstacle to making a diagnosis. On the contrary, while it is crying, you can better distinguish whether the cry is immediately under your ear or whether it is at some distance. When it is immediately under your ear, louder than normal, you conclude that some of the pulmonary tissue is condensed around the open bronchioles. Bronchophony can be heard often when bronchial respiration is not at all distinct. After studying a doubtful case for some time, you may find that the voice is increased on one side where perhaps but little dulness can be made out, the voice simply being increased on one side as compared with the other. That fact very often confirms the diagnosis. But we will not go into this subject to-day further than as it has a practical bearing on the present case.

We have here, then, a history of pertussis. Now, in cases of pertussis, just as in cases of measles, the occurence of lobular pneumonia is common. There are two reasons. First, because there is a general catarrh. Second, violent attacks of coughing. While the baby is coughing a good deal of mucus, the contents of the bronchial tubes is unable to get out, although some is expectorated. That which is in the trachea or large bronchi comes out during the violent coughing, while that which is in the smaller bronchi, particularly at the apices, is retained. Whooping-cough lasting so long, plenty of opportunity is offered for the development of pneumonia, and when it developes it is not likely to be soon recovered from. As long as the whooping-cough continues new pneumonic spots may form, so that the baby has practically one pneumonia after another. There is one peculiarity which will strike you in most of such cases, namely, that when the pneumonia is very extensive the character of the cough will change. The spasmodic attacks will sometimes cease, and when you see the case you may not make the diagnosis of whooping-cough, because the cough has rather the characteristics of a pneumonic than of a pertussis cough. But as soon as the pneumonia gets better the spasmodic character of the cough is liable to return. It is not only interesting, it is important to know that fact.

I have before spoken of belladonna in the treatment of whooping-cough. I should certainly recommend it here in full doses several times a day. It will act as a cardiac stimulant, while also acting against the whooping-cough. What has formerly been said about indications for treatment based on sleepless nights and wasting, applies in the present case. In most cases it is not necessary at all to give an antipyretic to reduce temperature, but it is necessary to give a cardiac tonic, no matter whether you select digitalis or strophanthus, or sparteine, or perhaps in some cases iodide of potassium. In some cases you will require, even in the acute stage of pneumonia, small doses of strychnia, camphor, or carbonate of ammonium. Do not forget what I have told you several times before, that we cannot always treat such cases according to the rules laid down in books. We must be guided by the individual case. The patient has to get well, not the disease. The patient has to live until he gets rid of the disease. Therefore, save his strength and do not wait until the symptoms of debility or heart failure have set in. While giving belladonna I would give some digitalis, and very probably some carbonate of ammonium with it. Keep the patient in an even temperature, 69° to 70° F., with a kettle of boiling water continually on the stove to moisten the air. At night the temperature might be a few degrees lower than during the day. But the fire should not be allowed to go out. A great deal depends upon the temperature of the room and the humidity of the air.—*Archives of Pediatrics*, Oct. 1893.

